# Effectiveness of Interventions to Reduce Potentially Inappropriate Medication in Older Patients: A Systematic Review

**DOI:** 10.3389/fphar.2021.777655

**Published:** 2022-01-24

**Authors:** Daniela A. Rodrigues, Ana I. Plácido, Ramona Mateos-Campos, Adolfo Figueiras, Maria Teresa Herdeiro, Fátima Roque

**Affiliations:** ^1^ Research Unit for Inland Development, Polytechnic Institute of Guarda (UDI-IPG), Guarda, Portugal; ^2^ Area of Preventive Medicine and Public Health, Department of Biomedical and Diagnostic Sciences, University of Salamanca, Salamanca, Spain; ^3^ Department of Preventive Medicine and Public Health, University of Santiago de Compostela, Santiago de Compostela, Spain; ^4^ Consortium for Biomedical Research in Epidemiology and Public Health (CIBER Epidemiology and Public Health-CIBERESP), Madrid, Spain; ^5^ Health Research Institute of Santiago de Compostela (IDIS), Santiago de Compostela, Spain; ^6^ Department of Medical Sciences, Institute of Biomedicine (iBiMED), University of Aveiro, Aveiro, Portugal; ^7^ Health Sciences Research Centre, University of Beira Interior (CICS-UBI), Covilhã, Portugal

**Keywords:** review, potentially inappropriate medication, interventions, effectiveness, older adults

## Abstract

**Background:** Age-related multiple comorbidities cause older adults to be prone to the use of potentially inappropriate medicines (PIM) resulting in an increased risk of adverse events. Several strategies have emerged to support PIM prescription, and a huge number of interventions to reduce PIM have been proposed. This work aims to analyze the effectiveness of PIM interventions directed to older adults.

**Methods:** A systematic review was performed searching the literature in the MEDLINE PubMed, EMBASE, and Cochrane scientific databases for interventional studies that assessed the PIM interventions in older adults (≥65 years).

**Results:** Forty-seven articles were included, involving 52 to 124,802 patients. Various types of interventions were analyzed such as medication review, educational strategies, clinical decision support system, and organizational and multifaceted approaches. In the hospital, the most successful intervention was medication review (75.0%), while in primary care, the analysis of all included studies revealed that educational strategies were the most effective. However, the analysis of interventions that have greater evidence by its design was inconclusive.

**Conclusion:** The results obtained in this work suggested that PIM-setting-directed interventions should be developed to promote the wellbeing of the patients through PIM reduction. Although the data obtained suggested that medication review was the most assertive strategy to decrease the number of PIM in the hospital setting, more studies are necessary.

**Systematic Review Registration:** [https://www.crd.york.ac.uk/prospero/display_record.php?ID=CRD42021233484], identifier [PROSPERO 2021 CRD42021233484].

## 1 Introduction

The increase in life expectancy associated with a declined birth rate contributed to rapid population aging (United Nations, 2019). Even though the world population is getting older, aging populations differ by region and level of development (Beard et al., 2016). Globally, it is estimated that in 2050 the number of older adults will reach 1.5 billion and will outnumber adolescents and youth aged 15–24 years (1.3 billion) (United Nations, 2019).

Considering that more than half of older adults have at least two chronic diseases (Barnett et al., 2012), these societal transformations pose a significant challenge in health systems and increase the consumption of health resources, including medicines. In addition, the treatment of chronic diseases is based on single disease-centered guidelines that can lead to an overwhelming of medication, and polypharmacy can easily occur (Barnett et al., 2012). Age-related pharmacokinetic and pharmacodynamic alterations associated with the use of multiple medicines can potentiate the consumption of potentially inappropriate medications (PIM) and facilitate the occurrence of adverse drug reactions (ADR) in frail older adults (Motter et al., 2018; Hefner et al., 2021).

PIM is defined as medicines that should not be prescribed because the risk of adverse events outweighs the clinical benefit, especially when more effective alternatives are available (Renom-Guiteras et al., 2015). The prescription of PIM has received special attention from the health community, and interventions aim to optimize medication prescribing and increase the benefit/risk ratio associated with the patients (Anderson et al., 1997; Simonson and Feinberg, 2005). In the last decades, several studies have been done to evaluate the effectiveness of PIM interventions in primary care, such as in hospitals and nursing homes. Nevertheless, the studies display widely differing methodology and inconsistent results, and to our knowledge, there are no systematic reviews comparing the effectiveness of different kinds of interventions.

Thus, this study aims to critically review the effectiveness of interventions to reduce PIM prescriptions in older adults.

## 2 Methods

This systematic review followed the PRISMA (Preferred Reporting Items for Systematic Reviews and Meta-Analyses) 2020 guidelines (Page et al., 2021) (Supplementary Table S1). The research protocol is registered on PROSPERO (CRD42021233484).

### 2.1 Search Strategy

A literature search was conducted in January 2021 and updated in February 2021 on the MEDLINE PubMed, EMBASE. A search was also conducted in the Cochrane database in October 2021. The search strategy was designed to identify relevant studies addressing interventions on PIM prescriptions in older adults, using the following broad-based search terms strategy: “(elderly OR “elderly patient” OR “older patient*” OR “older adult*” OR “geriatric patient*”) AND (PIM OR PIP OR “potentially inappropriate medicine” OR “potentially inappropriate medication” OR “potentially inappropriate prescribing” OR “prescribing patterns” OR “Prescription Drug Misuse” OR “Prescription Drug Overuse” OR “deprescri*” OR “potentially inappropriate prescription*“) AND (prevention OR reduction OR decrease OR impact) AND (intervention OR trial).”

### 2.2 Selection Criteria

This systematic review included the following: 1) all studies focused on PIM interventions directed to older adults (≥65 years) that aimed to optimize their pharmacotherapy; 2) controlled intervention studies and case series studies; and 3) all studies published in Portuguese, English, or Spanish between January 1, 2000, and December 31, 2020.

Excluded from this work were reviews, meta-analyses, opinions, letters to the editor that do not provide original data, comments, reports, studies addressing PIM in a specific pathology, and studies targeting a limited and predefined class of PIM.

### 2.3 Outcomes Measures

Our primary outcome measure was the effectiveness of the PIM interventions through the analysis of the change rate between the mean number of PIM per patient and/or the mean number of patients with PIM before and after an intervention.

### 2.4 Data Extraction

Two researchers (AP and DR) independently screened all titles and abstracts retrieved from the databases accordingly with the inclusion criteria. To evaluate the eligibility of full-text articles, two researchers (AP and DR) independently screened the full text of the articles. All discrepancies were resolved through discussion with the help of a third researcher (FR).

### 2.5 Quality Assessment

Two researchers (AP and DR) independently evaluated the quality and susceptibility to the bias of the included studies using the “Quality Assessment of Controlled Intervention Studies” and the “Quality Assessment Tool for Case Series Studies” tools, depending on study design (National Heart Laboratory, 2013). All discrepancies were resolved through discussion with a third (FR) or fourth researcher (MH).

### 2.6 Data Synthesis and Presentation

Two researchers (AP and DR) independently extracted data from the included studies. The data extracted from each article include authors, publication year, study design, country, sample size, patients' age, type of intervention applied, PIM screening tool, outcome measures, and main results.

To better analyze the extracted data, studies were grouped according to the intervention used. Within the intervention used, studies were grouped according to the setting where the intervention occurs. Five different interventions were identified in the included studies, and their descriptions were based on the following pre-defined definitions: 1) Medication review: “a structured evaluation of a patient's medicines to optimize medicines use and improve health outcomes. This entails detecting drug-related problems and recommending interventions” (Griese-Mammen et al., 2018); 2) educational interventions: “a package of interventions aimed to refresh the basic pharmacology competencies of a healthcare professional to change the prescription. The approaches used in the educational interventions included: interactive teaching, mailed educational material combined with individual feedback, and face-to-face visits to physicians” (Kaur et al., 2009); 3) clinical decision support systems (CDSS): “electronic tools that prompt provider behaviors in various areas of patient care, including medication ordering, chronic disease management, health care screening, and vaccination. CDSS can provide physicians, nurses, pharmacists, and other care providers with patient-specific prompts or warnings, treatment guidelines (e.g., order sets), automatic medication dosing calculators, or reports of overdue tests and medications as appropriate” (Bhugra and Cutter, 2001); 4) multifaceted interventions: “any intervention including two or more components.” In this study we classified as a multifaceted approach studies that used a combination of the interventions described above (Squires et al., 2014); and 4) organizational strategies: a combination of methodologies to improve the quality indicators. This type of intervention can include several methodologies, such as diagnostic activity, team building, intergroup relationship, sensitivity training, etc. In this work, organizational strategies include showing charts with the percentage of patients with PIM, an educational session with practices to identify patients with PIM, and frequent reunions to evaluate PIM indicators (Cady and Kim, 2017).

### 2.7 Statistical Analysis

A qualitative analysis was done if at least two studies had comparable outcomes. The heterogeneity of the studies was assessed through the comparison between the interventions. The efficacy of the interventions was presented as a change rate between the mean number of PIM per patient and/or the mean number of patients with PIM before and after an intervention.

## 3 Results

### 3.1 Study Selection

**FIGURE 1:**
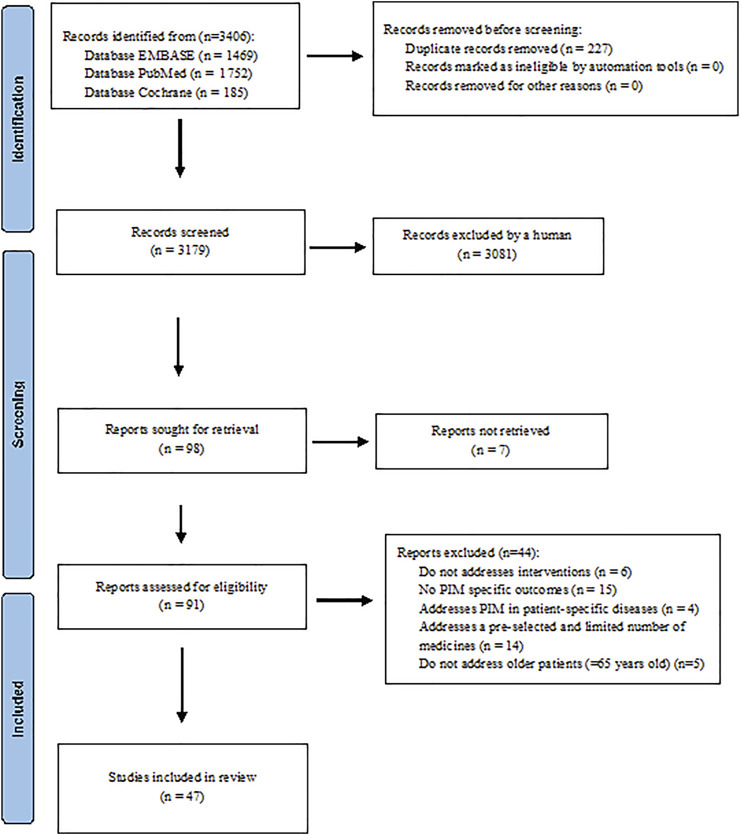
PRISMA diagram of the literature selection in this systematic review.

The search of the databases yielded 3,406 citations (Figure 1). After screening titles and abstracts, 98 articles potentially met the inclusion criteria. Because seven articles were not retrieved, only 91 articles were fully screened. Among these, 42 were excluded because they did not address interventions (*n* = 6), did not report PIM specific outcomes (*n* = 15), addressed PIM in patient-specific diseases (*n* = 4), addressed a pre-selected and/or a limited number of medicines (*n* = 14), and did not address older patients (≥65 years old) (*n* = 5) (Supplementary Table S2).

Forty-seven articles (Allard et al., 2001; Brown and Earnhart, 2004; Fick et al., 2004; Spinewine et al., 2007; Wessell et al., 2008; Castelino et al., 2010; Lampela et al., 2010; Gallagher et al., 2011; Keith et al., 2013; Rognstad et al., 2013; Dalleur et al., 2014; Franchi et al., 2014, 2016; Frankenthal et al., 2014; Lopatto et al., 2014; Clyne et al., 2015, 2016; Ilić et al., 2015; Tallon et al., 2015; Campins et al., 2016; Moss et al., 2016; Urfer et al., 2016; Frankenthal et al., 2017; Price et al., 2017; Stevens et al., 2017; Van der Linden et al., 2017; Vanderman et al., 2017; Chan et al., 2018; Etxeberria et al., 2018; Fajreldines et al., 2018; Gibert et al., 2018; Hurmuz et al., 2018; Najjar et al., 2018; Sennesael et al., 2018; Stuckey et al., 2018; Van Der Linden et al., 2018; Vandenberg et al., 2018; Boersma et al., 2019; Gutiérrez-Valencia et al., 2019; Khera et al., 2019; Liu et al., 2019; McDonald et al., 2019; Moss et al., 2019; Regueiro et al., 2019; Vu and Huong, 2019; Akkawi et al., 2020; Winata et al., 2020) fulfilled the inclusion criteria and were included in this systematic review (Figure 1).

### 3.2 Characteristics of Included Studies

A description of the characteristics of the included studies is presented in Table 1. Among the included studies, 24 were conducted in Europe (Spinewine et al., 2007; Lampela et al., 2010; Gallagher et al., 2011; Keith et al., 2013; Rognstad et al., 2013; Dalleur et al., 2014; Franchi et al., 2014; Lopatto et al., 2014; Clyne et al., 2015; Ilić et al., 2015; Tallon et al., 2015; Campins et al., 2016; Clyne et al., 2016; Franchi et al., 2016; Urfer et al., 2016; Van der Linden et al., 2017; Etxeberria et al., 2018; Gibert et al., 2018; Hurmuz et al., 2018; Sennesael et al., 2018; Van Der Linden et al., 2018; Boersma et al., 2019; Gutiérrez-Valencia et al., 2019; Regueiro et al., 2019), 14 in North America (Allard et al., 2001; Brown and Earnhart, 2004; Fick et al., 2004; Wessell et al., 2008; Moss et al., 2016; Price et al., 2017; Stevens et al., 2017; Vanderman et al., 2017; Chan et al., 2018; Stuckey et al., 2018; Vandenberg et al., 2018; Khera et al., 2019; McDonald et al., 2019; Moss et al., 2019), six in Asia (Frankenthal et al., 2014, 2017; Najjar et al., 2018; Liu et al., 2019; Vu and Huong, 2019; Akkawi et al., 2020), two in Oceania (Castelino et al., 2010; Winata et al., 2020), and one in South America (Fajreldines et al., 2018). The most frequented settings of the included studies were hospital (*n* = 26) (Brown and Earnhart, 2004; Spinewine et al., 2007; Gallagher et al., 2011; Dalleur et al., 2014; Franchi et al., 2014; Tallon et al., 2015; Franchi et al., 2016; Moss et al., 2016; Urfer et al., 2016; Stevens et al., 2017; Van der Linden et al., 2017; Vanderman et al., 2017; Chan et al., 2018; Fajreldines et al., 2018; Najjar et al., 2018; Sennesael et al., 2018; Van Der Linden et al., 2018; Vandenberg et al., 2018; Gutiérrez-Valencia et al., 2019; Liu et al., 2019; McDonald et al., 2019; Moss et al., 2019; Regueiro et al., 2019; Vu and Huong, 2019; Akkawi et al., 2020; Winata et al., 2020) and primary care (*n* = 18) (Allard et al., 2001; Fick et al., 2004; Wessell et al., 2008; Castelino et al., 2010; Lampela et al., 2010; Keith et al., 2013; Rognstad et al., 2013; Lopatto et al., 2014; Clyne et al., 2015; Campins et al., 2016; Clyne et al., 2016; Price et al., 2017; Etxeberria et al., 2018; Gibert et al., 2018; Hurmuz et al., 2018; Stuckey et al., 2018; Boersma et al., 2019; Khera et al., 2019). The number of participants in the studies ranged from 52 to 124,802. In the included studies, the average age of the participants ranged from 71 to 88.4 years. However, 12 studies did not report an average age of patients, although all of these studies provided an age range: 10 studies included older adults aged ≥65 years (Fick et al., 2004; Wessell et al., 2008; Moss et al., 2016; Frankenthal et al., 2017; Price et al., 2017; Stevens et al., 2017; Najjar et al., 2018; Vandenberg et al., 2018; Moss et al., 2019; Vu and Huong, 2019), one study included patients aged ≥70 years (Rognstad et al., 2013), and one study included patients aged ≥75 years (Lampela et al., 2010).

**TABLE 1:** Characteristics of the included studies (*n* = 47).

Author (year)	Country	Study design	Setting	Elderly patients' sample		Comparator	Quality assessment/(score obtained/total score)
				Sample size	Mean age (SD or IQR)		
Akkawi et al. (2020)	Malaysia	Case series	Hospital	B: 240	B: 71.9 (5.8)	Baseline	8/9^a^
				A: 240	A: 72.9 (5.7)		
Winata et al. (2020)	Australia	Case series	Hospital (aged care wards)	B: 121	B: 83.9 (7.2)	Baseline	8/9^a^
				A: 107	A: 83.3 (7.2)		
Boersma et al. (2019)	Netherlands	RCT	Geriatric clinic (outpatients)	C: 59	C: 79.0 (6.0)	Usual care	10/14^b^
				I: 65	I: 77.8 (5.7)		
Gutiérrez-Valencia et al. (2019)	Spain	Prospective study	Tertiary public hospital (acute geriatric unit)	234	87.6 (4.6)	Baseline	8/9^a^
Khera et al. (2019)	Canada	Quasi-experimental pretest–posttest	Primary care (community-dwelling patients)	54	81.7 (6.74, 65–95)	Before medication review	8/9^a^
Liu et al. (2019)	Taiwan	Interventional	Tertiary medical center (emergency department)	B: 243	B: 78.2 (7.7)	Before implementation of the intervention	8/9^a^
				A: 668	A: 78.1 (7.7)		
McDonald et al. (2019)	Canada	Non-randomized controlled before and after study	Medical clinical teaching units (internal medicine department)	C: 383	C: 79 (73–86)	Usual care	8/14^b^
				I: 417	I: 81 (74–88)		
Moss et al. (2019)	United States	Case series	Veteran Affairs Medical Center (emergency department)	C: 2,500	≥65	Untrained cohort	8/9^a^
				I: 3,162			
Regueiro et al. (2019)	Spain	Quasi-experimental pre–post	University hospital (internal medicine department)	174	82.6 (6.9)	Before implementation of the intervention	8/9^a^
Vu and Huong (2019)	Vietnam	Case series	General hospital (endocrinology, cardiology, and neurology departments)	B: 211	≥65	Baseline	8/9^a^
				A: 208			
Chan et al. (2018)	Canada	Retrospective single-center pre–post cohort	Tertiary hospital (acute care unit)	B: 70	B: 88.1 (4.3)	Before implementation of the intervention	7/9^a^
				A: 67	A: 88.4 (5.1)		
Etxeberria et al. (2018)	Spain	Case series	Primary health care	503	84.9 (3.8)	Before implementation of the intervention	7/9^a^
Fajreldines et al. (2018)	Argentina	Case series	Hospital	B: 640	B: 80.9 (9.8)	Before implementation of the intervention	8/9^a^
				A: 622	A: 79.3 (9.7)		
Hurmuz et al. (2018)	Netherlands	Retrospective longitudinal pretest vs. posttest	Community pharmacy	126	76.0 (7.4)	Before implementation of the intervention	8/9^a^
Najjar et al. (2018)	Saudi Arabia	Prospective pretest vs. posttest design	Hospital	B: 200	≥65	Baseline	6/9^a^
				A: 200			
Gibert et al. (2018)	France	—	Primary care	172	83.5 (4.9)	Before implementation of the intervention	8/9^a^
Sennesael et al. (2018)	Belgium	Retrospective interrupted time series study	Teaching hospital (geriatric unit)	120	85 (81–88)^c^	Standard geriatric care	7/9^a^
Stuckey et al. (2018)	United States	Prospective quality improvement project	Family medicine clinic (residency training outpatients)	34	74 (5)	Before implementation of the intervention	5/9^a^
Vandenberg et al. (2018)	United States	Quality improvement program	Veteran Affairs Medical Center (community-based outpatient clinic)	>7,000	≥65	Before implementation of the intervention	3/14^b^
Van Der Linden et al. (2018)	Belgium	Case series	Teaching hospital	B: 29	B: 83 (79–86^)^ ^c^	Usual care	8/14^b^
				A: 30	A: 83 (78–88)^c^		
Frankenthal et al. (2017)	Israel	RCT	Chronic care geriatric facility	C: 126	≥65	Usual care	7/14^b^
				I: 126			
Price et al. (2017)	Canada	RCT	Primary care	C: 1,086	≥65	Baseline rate	11/14^b^
				I: 1,204			
Stevens et al. (2017)	United States	—	Veteran Affairs Medical Center (emergency department)	—	≥65	—	9/9^a^
Vanderman et al. (2017)	United States	Retrospective cohort study	Veteran Affairs Medical Center (ambulatory clinics)	B: 1,539	B: 71.0 (6.72)	Usual care	7/9^a^
				A: 1,490	A: 71.0 (6.65)		
Van der Linden et al. (2017)	Belgium	Prospective controlled trial	Hospital (acute geriatric ward)	C: 81	C: 84.5 (4.97)	Usual care	8/14^b^
				I: 91	I: 84.5 (4.69)		
Campins et al. (2016)	Spain	RCT	Primary health care center	C: 251	C: 78.78 (5.46)	Routine clinical practice	12/14^b^
				I: 252	I: 79.16 (5.50)		
Clyne et al. (2016)	Ireland	RCT	Primary care	C: 97	C: 76.4 (4.8)	Usual care	11/14^b^
				I: 99	I: 77.1 (4.9)		
Franchi et al. (2016)	Italy	RCT	Hospital (internal medicine and geriatric wards)	C: 350	C: 83.8 (5.6)	Baseline	9/14^b^
				I: 347	I: 83.7 (5.9)		
Moss et al. (2016)	United States	—	Veteran Affairs Medical Center (emergency department)	23,168	≥65	—	6/9^a^
Urfer et al. (2016)	Switzerland	Case series	Hospital (internal medicine ward)	C: 450	C: 79 (73–84)	Patients hospitalized in some division	8/14^b^
				I: 450	I: 76 (71–83)		
Clyne et al. (2015)	Ireland	RCT	Primary care	C: 97	C: 76.4 (4.8)	Usual care	13/14^b^
				I: 99	I: 77.1 (4.9)		
Ilić et al. (2015)	Serbia	Case series	Nursing homes	104	82.6 (2.1)	Before implementation of the intervention	7/9^a^
Tallon et al. (2015)	Ireland	Case series	Teaching hospital	B: 60	B: 75 (70–80)	Standard care	7/9^a^
				A: 48	A: 78 (71–83)		
Dalleur et al. (2014)	Belgium	RCT	Teaching hospital	C: 72	C: 86 (81–89)	Usual care	8/14^b^
				I: 74	I: 84 (81–87)		
Franchi et al. (2014)	Italy	RCT	Hospital (internal medicine ward)	Admission C: 41; I: 40	Admission C85.58 (5.99), I: 82.8 (5.59)	Only the basic notions of pharmacology	9/14^b^
				Discharge C: 33; I: 37	Discharge, C: 80.92 (4.53), I: 82.49 (4.82)		
Frankenthal et al. (2014)	Israel	RCT	Chronic care geriatric facility	C: 176	82.7 (8.7)	Usual care	12/14^b^
				I: 183			
Lopatto et al. (2014)	Italy	—	Health authority database	111,282	75.29 (8.34)	-	7/9^a^
Keith et al. (2013)	Italy	Multi-phase prospective	Parma local health authority database	C: 81,597	C: 75.6 (7.3)	Region local health authority database	6/14^b^
				I: 78,482	I: 75.4 (7.2)		
Rognstad et al. (2013)	Norway	RCT	General practice	Control group	≥70	Baseline data	11/14^b^
				B: 35,073			
				After: 35,211 Intervention group			
				B: 46,737			
				A: 45,310			
Gallagher et al. (2011)	Ireland	RCT	Hospital (emergency department)	C: 192	C: 77 (71–81.75)	Usual care	13/14^b^
				I: 190	I: 74.5 (71–80)		
Castelino et al. (2010)	Australia	Retrospective	Primary care	372	76.1 (7.8)	Before implementation of the intervention	7/9^a^
Lampela et al. (2010)	Finland	RCT	Primary care	C: 500	≥75	Standard care	8/14^b^
				I: 500			
Wessell et al. (2008)	United States	Prospective	Primary care	124,802	≥65	—	6/9^a^
Spinewine et al. (2007)	Belgium	RCT	Teaching hospital	C: 90	C: 81.9 (6.2)	Usual care	10/14^b^
				I: 96	I: 82.4 (6.9)		
Brown and Earnhart (2004)	United States	Retrospective, case series	Teaching hospital	99	77.3	—	5/9^a^
Fick et al. (2004)	United States	RCT	Primary care	C: 185	≥65	Usual care	6/14^b^
				I: 170			
Allard et al. (2001)	Canada	RCT	Primary care	C: 130	C: 80.7 (4.6)	Usual care	10/14^b^
				I: 136	I: 80.4 (4.3)		

A, after; B, before; C, control group; I, intervention group; IQR, interquartile range; RCT, randomized controlled trial; SD, standard deviation.

a The National Institutes of Health (NIH) quality assessment tool for case series studies.

b The National Institutes of Health (NIH) quality assessment tool of controlled intervention study.

c Median age.

### 3.3 Quality of Included Studies

The quality assessment tools results of each study are reported in Table 2. Twenty-five articles fulfilled more than 80% of the exploratory questions (Castelino et al., 2010; Gallagher et al., 2011; Rognstad et al., 2013; Frankenthal et al., 2014; Lopatto et al., 2014; Clyne et al., 2015, 2016; Campins et al., 2016; Price et al., 2017; Stevens et al., 2017; Vanderman et al., 2017; Chan et al., 2018; Etxeberria et al., 2018; Fajreldines et al., 2018; Gibert et al., 2018; Hurmuz et al., 2018; Sennesael et al., 2018; Gutiérrez-Valencia et al., 2019; Khera et al., 2019; Liu et al., 2019; Moss et al., 2019; Regueiro et al., 2019; Vu and Huong, 2019; Akkawi et al., 2020; Winata et al., 2020). Eighteen studies pointed out clearly potential sources of bias (Brown and Earnhart, 2004; Fick et al., 2004; Spinewine et al., 2007; Wessell et al., 2008; Lampela et al., 2010; Rognstad et al., 2013; Campins et al., 2016; Franchi et al., 2016; Urfer et al., 2016; Frankenthal et al., 2017; Price et al., 2017; Van der Linden et al., 2017; Etxeberria et al., 2018; Gibert et al., 2018; Boersma et al., 2019; Gutiérrez-Valencia et al., 2019; Khera et al., 2019; Moss et al., 2019). Through analysis of Table 2, the main limitations were related to the low sample size and the lack of blinded intervention. Besides that, most of the studies did not do or report follow-up results, so it is not possible to understand if the interventions were effective in the middle/long-term.

**TABLE 2:** Quality assessment of included studies through the National Institutes of Health (NIH) quality assessment tools.

Quality assessment of controlled intervention studies				
No	Question	Number of studies (*n* = 22)		
		Yes	No	Other (CD, NA, NR)
1	Was the study described as randomized, a randomized trial, a randomized clinical trial, or an RCT?	16	6	0
2	Was the method of randomization adequate (i.e., use of randomly generated assignment)?	11	2	9
3	Was the treatment allocation concealed (so that assignments could not be predicted)?	10	3	9
4	Were study participants and providers blinded to treatment group assignment?	6	11	5
5	Were the people assessing the outcomes blinded to the participants' group assignments?	8	8	6
6	Were the groups similar at baseline on important characteristics that could affect outcomes (e.g., demographics, risk factors, co-morbid conditions)?	19	2	1
7	Was the overall drop-out rate from the study at endpoint 20% or lower of the number allocated to treatment?	13	9	0
8	Was the differential drop-out rate (between treatment groups) at endpoint 15 percentage points or lower?	19	1	2
9	Was there high adherence to the intervention protocols for each treatment group?	22	0	0
10	Were other interventions avoided or similar in the groups (e.g., similar background treatments)?	21	1	0
11	Were outcomes assessed using valid and reliable measures, implemented consistently across all study participants?	22	0	0
12	Did the authors report that the sample size was sufficiently large to be able to detect a difference in the main outcome between groups with at least 80% power?	12	4	6
13	Were outcomes reported or subgroups analyzed prespecified (i.e., identified before analyses were conducted)?	13	2	7
14	Were all randomized participants analyzed in the group to which they were originally assigned, i.e., did they use an intention-to-treat analysis?	9	0	13
**Quality assessment tool for case series studies**				
**No**	**Question**	**Number of Studies (*n* = 25)**		
		**Yes**	**No**	**Other (CD, NA, NR)**
1	Was the study question or objective clearly stated?	25	0	0
2	Was the study population clearly and fully described, including a case definition?	24	0	1
3	Were the cases consecutive?	4	6	15
4	Were the subjects comparable?	25	0	0
5	Was the intervention clearly described?	22	3	0
6	Were the outcome measures clearly defined, valid, reliable, and implemented consistently across all study participants?	22	3	0
7	Was the length of follow-up adequate?	4	0	21
8	Were the statistical methods described well?	21	4	0
9	Were the results described well?	24	1	0

### 3.4 Evidence of Effectiveness

#### 3.4.1 PIM Screening Tools

Thirty-seven studies used validated and published criteria to identify PIM, including Beers criteria (*n* = 16) (Brown and Earnhart, 2004; Fick et al., 2004; Wessell et al., 2008; Castelino et al., 2010; Lampela et al., 2010; Franchi et al., 2014; Franchi et al., 2016; Moss et al., 2016, 2019; Stevens et al., 2017; Vanderman et al., 2017; Chan et al., 2018; Stuckey et al., 2018; Vandenberg et al., 2018; Liu et al., 2019; Vu and Huong, 2019), Screening Tool of Older People's potentially inappropriate Prescriptions (STOPP) criteria (*n* = 15) (Gallagher et al., 2011; Dalleur et al., 2014; Frankenthal et al., 2014; Campins et al., 2016; Urfer et al., 2016; Frankenthal et al., 2017; Price et al., 2017; Fajreldines et al., 2018; Gibert et al., 2018; Hurmuz et al., 2018; Sennesael et al., 2018; Boersma et al., 2019; Gutiérrez-Valencia et al., 2019; Akkawi et al., 2020; Winata et al., 2020), a combination of Beers and STOPP criteria (*n* = 5) (Ilić et al., 2015; Etxeberria et al., 2018; Najjar et al., 2018; Khera et al., 2019; Regueiro et al., 2019), and Medication Appropriateness Index (MAI) (*n* = 1) (Tallon et al., 2015). Five studies used self-developed or adapted criteria (Allard et al., 2001; Keith et al., 2013; Lopatto et al., 2014; Van der Linden et al., 2017; Van Der Linden et al., 2018), and the remaining studies used a combination of validated (STOPP and/or Beers) and self-developed or adapted criteria.

#### 3.4.2 Interventions Used

After thorough analysis of the studies, five different types of interventions have emerged: medication review, educational interventions, CDSS, multifaceted approaches, and organizational strategies (Tables 3–7, respectively). Twenty-three studies used a medication review approach (Allard et al., 2001; Brown and Earnhart, 2004; Spinewine et al., 2007; Castelino et al., 2010; Lampela et al., 2010; Gallagher et al., 2011; Keith et al., 2013; Dalleur et al., 2014; Frankenthal et al., 2014; Lopatto et al., 2014; Tallon et al., 2015; Campins et al., 2016; Frankenthal et al., 2017; Van der Linden et al., 2017; Chan et al., 2018; Fajreldines et al., 2018; Hurmuz et al., 2018; Sennesael et al., 2018; Stuckey et al., 2018; Van Der Linden et al., 2018; Gutiérrez-Valencia et al., 2019; Khera et al., 2019; Regueiro et al., 2019), an educational intervention was the strategy used in eight studies (Fick et al., 2004; Rognstad et al., 2013; Franchi et al., 2014, 2016; Ilić et al., 2015; Clyne et al., 2016; Etxeberria et al., 2018; Moss et al., 2019), multifaceted approach was present in nine (Clyne et al., 2016; Moss et al., 2016; Gibert et al., 2018; Najjar et al., 2018; Vandenberg et al., 2018; Boersma et al., 2019; Liu et al., 2019; Vu and Huong, 2019; Akkawi et al., 2020) (i.e., a combination of different interventions), five studies used a CDSS (Urfer et al., 2016; Price et al., 2017; Vanderman et al., 2017; McDonald et al., 2019; Winata et al., 2020), and two used organizational strategies (Wessell et al., 2008; Stevens et al., 2017) (i.e., regulatory policies developed to decrease the number of PIM).

**TABLE 3:** Effects of medication review interventions on inappropriate prescribing in older adults (n = 23).

Author (year)	Performed by	PIM screening tool	Strategies used	Outcome measures	Significant outcomes
Gutiérrez-Valencia et al. (2019)	Pharm	STOPP criteria version 2	Pharmacist-led medicine optimization strategy	Difference in the number of patients with STOPP criteria and mean number of STOPP criteria per patient, before and after intervention	Patients with STOPP criteria
					B: 184 (78.6%) vs. A: 139 (59.4%), *p* < 0.001
					Mean number of STOPP criteria (SD)
					B: 1.8 (1.4) vs. A: 1.1 (1.2), *p* < 0.001
Khera et al. (2019)	Pharm	2015 Beers and version 2 of STOPP criteria	Pharmacist-led medication review	Number of medications satisfying explicit criteria of STOPP/Beers for PIM	Mean number of medications from STOPP/Beers criteria per patient (total sample) (SD)
					B. 1.15 (1.2) vs. A: 0.9 (1.1), *p* = 0.006
					Mean number of medications from STOPP/Beers criteria per patient (subjects with at least 1 PIM) (SD)
					B. 2.0 (0.97) vs. A: 1.6 (0.97), *p* = 0.005
Regueiro et al. (2019)	Investigator	Beers 2012 and STOPP 2008	PIM notification program	PIM number before and after	Not achieved
Chan et al. (2018)	Pharm	2015 Beers criteria	Collaborative medication reviews through a standardized template	Number of PIM that patients were taking at the time of admission and discharge	Not achieved
Fajreldines et al. (2018)	Pharm	STOPP criteria (2008)	Lectures and publications on STOPP criteria and suggestions made by clinical pharm to the physician on each individual prescription	Identification of PIM by the pharmacists before (on the admission and discharge) and after (admission and discharge) intervention	Patients with PIM on admission
					B: 48.9% vs. A: 47.4%
					Patients with PIM at discharge
					B: 46.1% vs. A: 16.7% *p* = 0.001
Hurmuz et al. (2018)	Pharm, Phys	STOPP criteria (version 2)	Medication reviews were initiated by the pharmacist and further carried out in close cooperation with the corresponding general practitioner	Number of PIM and appropriateness of prescribed medicines	Average number of PIM was initially 0.6 (SD = 0.8) per patient and decreased to 0.4, after the intervention (SD = 0.6, *p* < 0.05)
Sennesael et al. (2018)	Pharm	Short version of STOPP criteria (version 2)	Implementation of a screening tool in routine geriatric practice	Proportion of patients with ≥1 PIM	Not achieved
Stuckey et al. (2018)	Pharm, Phys	Beers criteria	Distribution of materials, multidisciplinary discussions, and computerized system	Total high-risk medications based on the Beers list	Total high-risk medications
					B: 42 vs. A: 28, *p* < 0.0005
Van Der Linden et al. (2018)	Investigators	RASP list	Systematic medication review	Number of RASP identified PIM at discharge; number of discontinued RASP PIM during hospital stay	Average number of RASP PIM at discharge (IQR)
					B: 2.5 (2.0–3.8) vs. A: 1 (0.0–3.0), *p* = 0.008
					Mean number of discontinued RASP PIM during hospital stay (SD)
					B: 0.79 (1.34) vs. A: 2.28 (1.62), *p* < 0.001
Frankenthal et al. (2017)	Study pharm	2008 STOPP criteria	Review of the medications by the study pharmacist	PIM proportion: number of residents with at least 1 PIM according to the STOPP criteria after 24 months	PIM according to STOPP criteria after 24 months
					C: 61 (48.4%) vs. I: 42 (33.3%), *p* = 0.02
Van der Linden et al. (2017)	Pharm	RASP list	Pharmacist-led medication review and recommendations reported to the treating physician daily	Number of RASP PIM, proportion of discontinued or reduced drugs that was identified by the RASP list	Average number of discontinued or reduced drugs identified by the RASP list (IQR)
					C: 1 (1–2) vs. I: 2 (1–4), *p* = 0.003
Campins et al. (2016)	Pharm	STOPP criteria (version 2)	A pharmacist evaluated all drugs prescribed to each patient and discussed recommendations for each drug with the patient's physician and then with the patient. A final decision was agreed by physicians and their patients in a face-to-face visit	Proportion of prescriptions rated as PIM; rate of acceptance by physicians	Not achieved
Tallon et al. (2015)	Pharm	MAI	Collaborative PACT model on the medication appropriateness of acute hospitalized older patients	Appropriateness of prescribing at pre-admission, during admission, and at discharge	PACT significantly improved the MAI score from pre-admission to admission (mean difference 2.4, 95% CI 1.0 to 3.9, *p* < 0.005) and from pre-admission to discharge (mean difference 4.0, 95 CI 1.7 to 6.4, *p* < 0.005)
					PACT resulted in significantly fewer drugs with 1 or more inappropriate rating at discharge (PACT 15.0%, standard 30.5%, *p* < 0.001)
Dalleur et al. (2014)	Ger	STOPP criteria (2008)	STOPP criteria recommendations from an inpatient geriatric consultation team (IGCT)	Proportion of PIM discontinued	Discontinuation at discharge of PIM present on admission
					C: 19.3% vs. I: 39.7%, *p* = 0.013
Frankenthal et al. (2014)	Study pharm	STOPP criteria (2008)	Medication review for all residents at study opening and 6 and 12 months later based on STOPP criteria	Number of PIM over time	Number of PIM at baseline
					C: 114 (64.7%) vs. I: 129 (70.5%)
					Number of PIM after 6-month follow-up
					C: 89 (56%) vs. I: 65 (37.4%), *p* = 0.001
					Number of PIM after 12-month follow up
					C: 79 (54.1%) vs. I: 36 (22.5%), *p* < 0.001
Lopatto et al. (2014)	Phys	Maio criteria	Participatory clinical guidelines development, group educational outreach, and dissemination of educational materials combined with peer-to-peer interactive discussion	PIM incidence rate	Not achieved
Keith et al. (2013)	Phys	Maio criteria	Participatory clinical guidelines development, group educational outreach, and dissemination of educational materials combined with peer-to-peer interactive discussion	Quarterly incidence rates of older patients exposed to PIM	Patients exposed to at least 1 PIM
					2007
					C: 6,315 (7.7%) vs. I: 6,098 (7.7%)
					2009
					C: 5,111 (6.1%) vs. I: 4,277 (5.3%) *p* < 0.001
Gallagher et al. (2011)	Phys	2008 STOPP criteria	STOPP screening and recommendations to the attending medical team	Patients with ≥1 STOPP criteria at discharge	Patients with ≥1 STOPP criteria at discharge
					C: 93 (48.4%); I: 7 (3.7%), *p* < 0.001
Castelino et al. (2010)	Pharm	2003 Beers criteria	Home Medicine Review (HMR) service	Rate of PIM	Not achieved
Lampela et al. (2010)	Phys, N, physiotherapist, nutritionist	1997 Beers criteria (US 2003 update)	Adjustment of a patient's medication when necessary; evaluation of the indications for all drugs in use; clinical examination, including careful evaluation of cognition, mood, orthostatic reactions, and presence of extrapyramidal symptoms; routine blood tests	Numbers of inappropriate drugs or dosages	Not achieved
Spinewine et al. (2007)	Pharm	MAI, Beers (1997), and ACOVE criteria	The appropriateness of treatment was analyzed, and a pharmaceutical care plan was prepared. Whenever an opportunity for optimization was identified, the pharmacist discussed that opportunity with the prescriber, who could accept or reject the intervention	Appropriateness of prescribing at admission, discharge, and 3 months after discharge using Beers' criteria	Intervention patients significantly more likely than control patients to have improvements in Beers' criteria [OR 0.6 (95% CI 0.3, 1.1)]
Brown and Earnhart (2004)	Pharm	Beers criteria (1997)	Acute Care for Elders (ACE) team improvement on the medication regime of geriatric inpatients	Prevalence of PIM	Rate of PIM at admission 10.1%, and discharge 2.02%, *p* < 0.02
Allard et al. (2001)	Pharm, N, Phys	List of PIM developed by the Quebec Committee on Drug Use in the Elderly	A team comprising 2 physicians, a pharmacist, and a nurse reviewed the list of drugs and the diagnoses of patients and formulated suggestions that were mailed to the patients' physician	Number of PIM; number of subjects with at least 1 PIM	Not achieved

A, after group; B, before group; C, control group; CI, confidence interval; Ger, geriatrician; I, intervention group; IQR, interquartile range; MAI, Medication Appropriateness Index; N, nurse; Pharm, pharmacist; Phys, physician; PACT, pharmaceutical care at Tallaght Hospital; PIM, potentially inappropriate medication; RASP, Rationalization of Home Medication by an Adjusted STOPP list in Older Patient; SD, standard deviation; STOPP, Screening Tool of Older People's potentially inappropriate Prescriptions; US, United States.

**TABLE 4:** Effects of educational interventions on inappropriate prescribing in older adults (*n* = 8).

Author (year)	Performed by	Receivers	PIM screening tool	Strategies used	Outcome measures	Significant outcomes
Moss et al. (2016)	Pharm, Phys	Medical residents	Table 2 of 2012 Beers criteria	Enhancing Quality of Prescribing Practices for Veterans Discharged from the Emergency Department (EQUiPPED) provider education through academic detailing, clinical decision support, and provider feedback on prescribing practices	Prescription rate ratio before and after the intervention	The group after the intervention were less likely to prescribe a PIM when compared to the group before the intervention (rate ratio = 0.73, 95% CI = 0.632–0.850; *p* < 0.0001)
Etxeberria et al. (2018)	Research team	Phys	STOPP and Beers criteria	Electronic identification of PIM, training for physicians and structured review of medication	Change in the number of PIM per patient	Number of PIM/patients (SD)
						B: 0.70 (0.91) vs. A: 0.51 (0.77), *p* < 0.0001
Clyne et al. (2016)	Pharm	Phys (C: 10, I: 11)	Beers, STOPP, McLeod, IPET, ACOVE, and the Prescription Peer Academic Detailing (RxPAD) study—MRC framework	Academic detailing, review of medicines with web-based pharmaceutical treatment algorithms that provide recommended alternative-treatment options, and tailored patient information leaflets	Proportion of patients with PIM and mean number of PIM	Mean number of PIM (SD)
						C: 1.03 (0.8) vs. I: 0.61 (0.7), *p* = 0.01
Franchi et al. (2016)	Research team	Phys	2012 Beers criteria	E-learning educational program	Reduction in the PIM prescriptions at hospital discharge (at least 1 PIM)	Not achieved
Ilić et al. (2015)	Investigator (Phys)	27 Phys	2012 Beers criteria and 2008 STOPP criteria	Lectures and brochures	Inappropriately prescribed drugs	Average number of PIM (Beers) (IQR)
						B: 11.0 (1.0–43.0) vs. A: 1.0 (1.0–2.0), *p* < 0.001
						Average number of PIM (STOPP) (IQR)
						B: 3.5 (1.0–20.0) vs. A: 1.5 (0.0–6.0), *p* < 0.005
Franchi et al. (2014)	Research Team	Phys (C: 22, I: 54)	2012 Beers criteria	E-learning educational program	Reduction of prescription of PIM	Not achieved
Rognstad et al. (2013)	Phys (peer academic detailers)	Phys (C: 209, I: 256)	13 explicit PIM criteria, assumed to be relevant for the Norwegian general practice setting (based on Beers criteria and The Swedish National Board of Health and Welfare)	Multifaceted educational intervention with feedback and audit	Changes in prescription patterns	Not achieved
Fick et al. (2004)	Research team	Phys (C: 185, I: 170)	1997 Beers criteria for medications to avoid in older adults	Integrated decision support service: 1) a detailed educational brochure listing PIM, 2) a list of suggested PIM alternative medications, and 3) a personally addressed letter that described in detail all the physician's patients who were determined to be in receipt of 1 or more PIM	Rate of providers that prescribed at least 1 PIM	Number of continuously enrolled members with at least 1 PIM declined significantly (*χ*2 = 13.20, *p* < 0.001) to 17.9% (3,007/16,818), from a baseline of 19.4% (3,364/17,330)

A, after group; B, before group; C, control group; I, intervention group; Pharm, pharmacist; Phys, physician; PIM, potentially inappropriate medication; SD, standard deviation; STOPP, Screening Tool of Older People's potentially inappropriate Prescriptions.

**TABLE 5:** Effects of clinical decision support system (CDSS) interventions on inappropriate prescribing in older adults (*n* = 5).

Author (year)	PIM screening tool	Strategies used	Outcome measures	Significant outcomes
Winata et al. (2020)	STOPP version 2	Introduction of an electronic medication management system (EMMS)	Number of PIM on admission and discharge per patient; number of patients with ≥1 PIM on admission and discharge	Not achieved
McDonald et al. (2019)	Beers and STOPP criteria (version 2), and Choosing Wisely lists	Electronic decision support tool that generates deprescribing opportunities reports	Proportion of patients with 1 or more home medications identified as a PIM and deprescribed at hospital discharge	Not achieved
Price et al. (2017)	STOPP criteria	Electronic medical record with automated STOPP rules	Change in measured PIM rates between the intervention and control groups before the intervention as compared with the difference after the intervention period	Not achieved
Vanderman et al. (2017)	2012 Beers criteria	Medication alert message	Overall PIM, top 10 PIM, and flagged PIM	New top 10 PIM/new total medications
				B: 1,405/15,539 (12.56%) vs. A: 1,308/15,807 (12.00%), *p* = 0.0158
Urfer et al. (2016)	2008 STOPP criteria	Easy-to-use 5-point checklist: 1) ascertain all current medications used; 2) identify patients at high risk of adverse drug reactions; 3) estimate life expectancy; 4) identify medications which are not indicated and/or are potentially dangerous; and 5) monitor the patient if drugs were stopped or new drugs were added	Proportion of patients prescribed PIM at discharge	Patients with >1 PIM at discharge
				B: 164 (39.0%) vs. A: 102 (23.7%), *p* < 0.001

A, after group; B, before group; PIM, potentially inappropriate medication; STOPP, Screening Tool of Older People's potentially inappropriate Prescriptions.

**TABLE 6:** Effects of multifaceted interventions on inappropriate prescribing in older adults (*n* = 9).

Author (year)	Educational		CDSS	Medication review	PIM screening tool	Strategies used	Outcome measures	Significant outcomes
	Performed by	Receivers						
Akkawi et al. (2020)	Not reported	Phys, Pharm	X		STOPP version 2	Three educational sessions about PIM and discussion of STOPP criteria, coupled with an introduction of a CDSS	PIM prevalence	Not achieved
Boersma et al. (2019)			X	Phys	STOPP version 1	The intervention consisted of written prescribing recommendations prepared by an independent, clinically experienced research physician using the STRIP Assistant	PIM changes implementation	PIM changes implementations
								C: 15.3% vs. I: 46.2% (*p* < 0.001)
Liu et al. (2019)			X	Pharm, Phys	Modified and updated 2015 Beers criteria according to common practice and culture in Taiwan	Creation of a multidisciplinary Chi-Mei Integrated Geriatric Emergency Team; creation of a PIM list; computer-based medication reconciliation and integration system to obtain information about medications prescribed	Number of PIM at hospital admission and discharge	Number of PIM on admission
								B: 173 vs. A: 480, and at discharge
								B: 88 vs. A: 156. *p* < 0.001
Vu and Huong (2019)	Pharm	Phys		Pharm	2015 Beers criteria	Pharmacists gave a training lecture on the Beers 2015 criteria in 2 h for the medical doctors; the training was also conducted as face-to-face visits. The notebook with the Beers 2015 criteria was provided to the medical doctors	Prevalence of PIM	Prevalence of PIM
								B: 34.1% vs. A: 23.1%, (odds ratio (OR) = 0.337, 95% CI = 0.207–0.551, *p* < 0.001)
Najjar et al. (2018)	Head of geriatric medicine, 2 Pharms	Pharm		Pharm, Phys	STOPP (version 2) and Beers (2015) criteria	Educational program consisting of 1-h, weekly educational lectures for 1 month, handbook designed was distributed to the physicians at the end of the seminars; collaboration between clinical pharmacists and the prescribers to optimize prescribing: auditing of the physician's orders and providing feedback and recommendations during medical rounds, reminders, and discussions with physicians	Change in the incidence rate of PIM	Incidence rate of PIM
								B: 61% vs. A: 29.5%, *p* < 0.001
Gibert et al. (2018)	Research team	20 Phys		Phys	STOPP criteria	STOPP criteria use during primary care GP consultations	Proportion of patients with a reduction of PIM after the intervention	This intervention reduced PIM for 44.9% of the patients (*n* = 44), *p* < 0.001
Vandenberg et al. (2018)	Geriatrician, Pharm, Gerontologist	20 primary care providers, 4 Pharms		Pharm	2012 Beers criteria	A pharmacist-led, one-on-one medication review, to provide rural primary care providers and pharmacists with educational outreach through academic detailing and tools to support safe geriatric prescribing practices, as well as individual audit and feedback on prescribing practice and confidential peer benchmarking	PIM incidence: number of new PIM prescriptions divided by all encounters (opportunities) that a provider had with veterans aged 65 years and older; PIM prevalence—number of encounters with veterans currently taking at least 1 PIM divided by all encounters; multiple PIM prevalence—number of encounters with veterans taking 2 or more PIM divided by all encounters	the intervention, reaching significance (*p* = 0.009) during the postintervention period. PIM prevalence declined at baseline, 22.6% encounters per month were with older veterans taking at least 1 PIM. After the intervention, this proportion had dropped to 16.7% (*p* < 0.001), a 26% relative reduction. Encounters with veterans taking 2 or more PIM showed steady and significant decline, from a baseline of 6.2–4.1% after the intervention (*p* < 0.001), representing a 33.9% relative reduction in multiple PIM prevalence
Moss et al. (2016)	Phys, Geriatricians, Gerontologists, Pharm, N, Clinical application coordinators	73 ED providers (10 physicians, 60 medical residents), 3 advanced practice providers	X		2012 Beers criteria	Provider education; clinical decision support, and provider feedback on prescribing practices	Rate of PIM prescribing over the observation period	Not achieved
Clyne et al. (2015)	Pharm	Phys (C: 11, I: 10)		Phys	Beers, STOPP, McLeod, IPET, ACOVE, and the Prescription Peer Academic Detailing (RxPAD) study—MRC framework	Academic detailing, review of medicines with web-based pharmaceutical treatment algorithms that provide recommended alternative-treatment options, and tailored patient information leaflets	Mean number of PIM	Mean number of PIM (SD) at baseline
								C: 1.39 (0.6) vs. I: 1.31 (0.6)
								Mean number of PIM (SD) after intervention completed
								C: 1.18 (0.1) vs. I: 0.70 (0.1), *p* = 0.02

A, after group; B, before group; C, control group; CDSS, clinical decision support system; CI, confidence interval; ED, emergency department; GP, general practitioner; I, intervention group; N, nurse; Pharm, pharmacist; Phys, physician; PIM, potentially inappropriate medication; SD, standard deviation; STRIP, systematic tool to reduce inappropriate prescribing; STOPP, Screening Tool of Older People's potentially inappropriate Prescriptions.

#### 3.4.3 Impact of Medication Review Interventions

Medication review was conducted by different healthcare professionals: physicians (Gallagher et al., 2011; Keith et al., 2013; Lopatto et al., 2014), pharmacists (Brown and Earnhart, 2004; Spinewine et al., 2007; Castelino et al., 2010; Frankenthal et al., 2014; Tallon et al., 2015; Campins et al., 2016; Van der Linden et al., 2017; Chan et al., 2018; Fajreldines et al., 2018; Sennesael et al., 2018; Gutiérrez-Valencia et al., 2019; Khera et al., 2019), pharmacists and physicians (Hurmuz et al., 2018; Stuckey et al., 2018), gerontologists (Dalleur et al., 2014), investigators (Van Der Linden et al., 2018; Regueiro et al., 2019), pharmacist students (Frankenthal et al., 2017), and multidisciplinary teams (Allard et al., 2001; Lampela et al., 2010). Although medication review intervention varies in the included studies, this intervention involved the analysis of the patient's pharmacotherapeutic needs and prescribed drugs, followed by a recommendation to optimize medication.

Six (Brown and Earnhart, 2004; Spinewine et al., 2007; Tallon et al., 2015; Van der Linden et al., 2017; Fajreldines et al., 2018; Gutiérrez-Valencia et al., 2019) of eight medication review interventions conducted by pharmacists at the hospital (Brown and Earnhart, 2004; Spinewine et al., 2007; Tallon et al., 2015; Van der Linden et al., 2017; Chan et al., 2018; Fajreldines et al., 2018; Sennesael et al., 2018; Gutiérrez-Valencia et al., 2019) demonstrate a positive impact on PIM reduction. Among these, in two studies the intervention improved the PIM screening tool score (Spinewine et al., 2007; Tallon et al., 2015). Fajreldines et al. (2018) and Gutiérrez-Valencia et al. (2019) reported that the number of patients with PIM significantly decreased after medication review (19.2% and 30.7%, respectively). Van der Linden et al. (2017) observed a decrease in the average number of PIM after an intervention. Finally, Brown and Earnhart (2004) conclude that their intervention led to an 8.08% absolute risk reduction.

Among the studies performed by physicians in hospitals, one was conducted at an emergency department of a hospital (Gallagher et al., 2011), and the remaining two studies used the local health authority databases (Keith et al., 2013; Lopatto et al., 2014). Gallagher et al. (2011) observed a significant reduction in the proportion of patients with at least one PIM in the intervention group (from 43.2% in admission to 3.7%, at discharge). This trend remains stable during the 6 months of follow-up. One study performed a quality improvement program across an Italian region with more than 80,000 older adults and observed that the PIM exposure incidence rate significantly declined 31.4% (from the baseline to the post-intervention period) (Keith et al., 2013).

The intervention performed by a gerontologist in hospitalized patients results in a significant decrease in the number of PIM in patients discharged (PIM discontinuation of 39.7%) (Dalleur et al., 2014). Similar outcomes were achieved in the intervention performed by investigators in hospitalized patients (Van Der Linden et al., 2018).

In primary care, the medication review performed by both physicians and pharmacists results in a significant reduction in the mean number of PIM per patient [from 0.6 to 0.4 (Hurmuz et al., 2018) and from 1.24 to 0.82 (Stuckey et al., 2018)]. In this setting, a multidisciplinary team failed to achieve a significant reduction in the number of PIM, and only one (Khera et al., 2019) of the three pharmacists' interventions (Castelino et al., 2010; Campins et al., 2016; Khera et al., 2019) results in a significant impact in PIM reduction.

Finally, in a chronic care geriatric facility, pharmacy students observed a decline in PIM prescriptions after a follow-up of 12 and 24 months (Frankenthal et al., 2014; Frankenthal et al., 2017).

#### 3.4.4 Impact of Educational Interventions

In the eight included studies (Fick et al., 2004; Rognstad et al., 2013; Franchi et al., 2014, 2016; Ilić et al., 2015; Clyne et al., 2016; Etxeberria et al., 2018; Moss et al., 2019) that used educational approaches to reduce PIM, the interventions were performed by the following: a researcher and/or a research team (Fick et al., 2004; Franchi et al., 2014, 2016; Ilić et al., 2015; Etxeberria et al., 2018), a multifaceted team containing a pharmacist and a physician (Moss et al., 2019) or physicians (peer academic detailers) (Rognstad et al., 2013), or a pharmacist (Clyne et al., 2016). In all studies the target of the educational interventions were physicians. The outcomes of the interventions were measured through the reduction of PIM use or PIM prescriptions. In one of the studies, the included population was polymedicated older adults (Etxeberria et al., 2018); in the three studies that have a positive impact on PIM, the average number of PIM per patient ranged from 0.7–11 before intervention to 0.51–1.5 after the intervention (Ilić et al., 2015; Clyne et al., 2016; Etxeberria et al., 2018). One study reported that the number of physicians that prescribed at least one PIM declined 17.9% (Fick et al., 2004). Finally, one study reported that after the educational intervention the PIM rate ratio before and after the intervention is 0.73 (Moss et al., 2019).

#### 3.4.5 Impact of Clinical Decision Support System Interventions

Five studies used CDSS to reduce PIM (Urfer et al., 2016; Price et al., 2017; Vanderman et al., 2017; McDonald et al., 2019; Winata et al., 2020). Two of four studies performed in hospitalized patients reported that the implementation of CDSS has a positive impact on PIM deprescription (Urfer et al., 2016; Vanderman et al., 2017). In one study, the introduction of a PIM checklist, in an internal medicine ward, leads to a significant reduction (22.0%) of the risk of being prescribed one or more PIM (Urfer et al., 2016). Finally, one study observed that although the total number of newly prescribed PIM did not decrease, the top 10 most common new PIM significantly decreased from 9.0% to 8.3% (Vanderman et al., 2017).

#### 3.4.6 Impact of Multifaceted Interventions

Nine studies used a multifaceted approach as a strategy to decrease the number of PIM (Clyne et al., 2016; Moss et al., 2016; Gibert et al., 2018; Najjar et al., 2018; Vandenberg et al., 2018; Boersma et al., 2019; Liu et al., 2019; Vu and Huong, 2019; Akkawi et al., 2020) In two studies, the multifaceted approach consisted in the application of a CDSS followed by a medication review (Boersma et al., 2019; Liu et al., 2019). One study used a combination of educational and CDSS approaches (Akkawi et al., 2020). The remaining studies used, as an approach to decrease the number of PIM, a combination of an educational approach followed by a medication review (Clyne et al., 2016; Moss et al., 2016; Gibert et al., 2018; Najjar et al., 2018; Vandenberg et al., 2018; Vu and Huong, 2019).

##### Clinical Decision Support System and Medication Review

The combined use of CDSS and medication review strategies led to a significant reduction of the mean number of PIM per patient from 0.7 to 0.23 (Boersma et al., 2019) and an increasing number of PIM changes implementation (intervention: 46.2% vs. control: 15.3%) (Liu et al., 2019).

##### Educational Intervention and Medication Review

Five of the six studies that used a combination of educational and medication review strategies observed a significant impact on the PIM reduction (Clyne et al., 2016; Gibert et al., 2018; Najjar et al., 2018; Vandenberg et al., 2018; Vu and Huong, 2019). Among these, two studies reported a significant decrease in the mean number of PIM per patient from 0.99–1.18 before intervention to 0.66 to 0.7, after intervention (Clyne et al., 2015; Gibert et al., 2018). Vandenberg et al. (2018) and Vu and Huong (2019) reported that the prevalence of PIM decreased 5.9% and 10.9%, respectively. According to Najjar et al. (2018), the multifaceted approach led to a decrease in the PIM incidence of 31.5%.

##### Educational Intervention and Clinical Decision Support System

In one study (Akkawi et al., 2020), the multifaceted intervention consists of three educational sessions about PIM and discussion of STOPP criteria, coupled with an introduction of a CDSS. This approach did not achieve significant outcomes.

#### 3.4.7 Impact of Organizational Interventions

One study performed an organizational intervention in four different hospitals and observed a significant decrease in the average percentage of prescribed PIM per month (1.7–6.8) after intervention (Stevens et al., 2017). Another study performed in 99 primary care practices observed that the organizational intervention that includes PIM performance reports, on-site visits, and network meetings was responsible for an absolute annual decline of 0.018% for always inappropriate medication (Wessell et al., 2008).

**TABLE 7:** Effects of organizational interventions^1^ on inappropriate prescribing in older adults (*n* = 2).

Author (year)	PIM screening tool	Strategies used	Outcome measures	Significant outcomes
Stevens et al. (2017)	2012 Beers criteria	Education, informatics-based clinical decision support designed for improved workflow, and individual provider feedback	Average percentage of PIM	Average percentage of PIM per month (SD): Site 1 – B: 11.9% (1.8) vs A: 5.1% (1.5), *p* < 0.0001 Site 2 – B: 8.2% (0.8) vs A: 4.5% (1.0), *p* < 0.0001 Site 3 – B: 8.9% (1.9) vs A: 6.1% (1.7), *p* = 0.0007 Site 4 – B: 7.4% (1.7) vs A: 5.7% (0.8), *p* = 0.04
Wessell et al. (2008)	1997 Beers criteria (US 2003 update)	A quarterly PIM performance report; biannual on-site visits	Change in the prescription rate	Absolute annual decline of 0.018% for always inappropriate medications (*p* = 0.03)

A, After group; B, Before group; PIM, Potentially inappropriate medication; SD, Standard deviation; US, United States.

1 Organizational intervention- a combination of strategies to improve the quality indicators of institutions/organizations and enrolled in the approach all stakeholders, health professionals, and non-health professionals. This intervention uses several approaches, including diagnostic activity (including medication review), Team Building, Intergroup relationship, sensitivity training (including educational sessions.

#### 3.4.8 Impact of Interventions That Have Greater Evidence by Its Design

The analysis of the studies that included a concurrent control and low risk of bias revealed that in the hospital, all the five medication review interventions were effective (Spinewine et al., 2007; Gallagher et al., 2011; Dalleur et al., 2014; Van der Linden et al., 2017; Van Der Linden et al., 2018), two CDSS interventions were effective (Urfer et al., 2016; McDonald et al., 2019), one multifaceted intervention achieved significant impact (Vandenberg et al., 2018), and none of the educational interventions achieved a successful reduction of PIM. In primary care it was observed that all the two multifaceted interventions achieved a significant reduction of PIM (Clyne et al., 2015; Boersma et al., 2019), one (Clyne et al., 2016) of two educational strategies was effective, and none of the medication review and CDSS strategies was well successful.

## 4 Discussion

Despite the extensive number of studies in the literature on PIM in older patients, only 31 of the included studies reported effective intervention. Among these, 21 presented methodological intervention limitations and could not ensure that the intervention used can be replicated and identical outcomes achieved (Brown and Earnhart, 2004; Fick et al., 2004; Spinewine et al., 2007; Wessell et al., 2008; Gallagher et al., 2011; Dalleur et al., 2014; Frankenthal et al., 2014; Clyne et al., 2015; Tallon et al., 2015; Urfer et al., 2016; Frankenthal et al., 2017; Van der Linden et al., 2017; Etxeberria et al., 2018; Gibert et al., 2018; Hurmuz et al., 2018; Van Der Linden et al., 2018; Boersma et al., 2019; Gutiérrez-Valencia et al., 2019; Khera et al., 2019; Liu et al., 2019; Vu and Huong, 2019). Although a meta-analysis was not done, our findings suggested that in the hospital, the most adequate strategy to decrease the number of PIM and/or the patients with at least one PIM was medication review. Concerning primary care setting, the analysis of all the included studies indicated that educational interventions were the most successful. However, when only randomized controlled trial (RCT) studies were analyzed, it did not find greater effectiveness of some interventions over others.

The data of this study also suggested that the inclusion of pharmacists can upgrade the quality of the PIM intervention and effectively promote the well-being of the patients.

Regarding the influence of the number of prescribed medicines per patient in PIM interventions, our data suggested that the success of an intervention is not medicines number-dependent, since the analysis of the successful intervention rate in polymedicated and non-polymedicated patients was similar (≈67%).

This work also suggested that most of the studies presented important design limitations, something that limits the grade of their evidence.

Medication review was the most frequent strategy used to improve pharmacotherapy and reduce the number of PIM in hospitalized patients. A reduction in the number of PIM per patient or/and in the number of patients with at least a PIM was achieved for 75% of the medication review interventions (Brown and Earnhart 2004; Spinewine et al., 2007; Gallagher et al., 2011; Keith et al., 2013; Dalleur et al., 2014; Ilić et al., 2015; Tallon et al., 2015; Van der Linden et al., 2017; Chan et al., 2018; Fajreldines et al. 2018; Van Der Linden et al., 2018; Gutiérrez-Valencia et al., 2019). Among the three studies that do not have efficacy in hospitalized patients, the main reasons pointed were as follows: 1) the difficulty to engage physicians to actively participate in the study—they preferred to receive the documentation about drug therapy issues by paper instead of discussing face-to-face the patients' pharmacotherapy (Chan et al., 2018); and 2) the low acceptance of the recommendation by the physicians (Regueiro et al., 2019).

In primary care, 42.9% of the interventional studies (Keith et al., 2013; Hurmuz et al., 2018; Stuckey et al., 2018; Khera et al., 2019) used medication review to improve the pharmacotherapy through the reduction of PIM. The lack of efficacy can be related to 1) the low acceptance rate of recommendations by the physicians (Allard et al., 2001); 2) the PIM list used—for example, Castelino et al. reported that in Australia the medicines listed in Beers criteria were rarely used; 3) the physicians did not use routinely any checklist and do not access computer programs to evaluate hypothetical interactions, and they do not record short-term drug alterations (Lampela et al., 2010); 4) in some cases the patients did not receive the full intervention (Allard et al., 2001); and 6) contamination between control and intervention groups (Campins et al., 2016).

The analysis of all educational interventions performed in primary care revealed that this type of intervention has been successfully implemented in 75%. However, only one of the two studies that have greater evidence by its design effectively decreased the number of PIM. The success of educational interventions in primary care can be related to the promotion of a specific web training on PIM tools used by physicians in clinical practice, updating the knowledge of physicians in PIM detection (Fick et al., 2004; Clyne et al., 2016; Etxeberria et al., 2018). The lack of efficacy of educational intervention observed in one study can be related to a change in the participants' behavior due to the knowledge that they are taking part in an experiment (Hawthorne effect) (Rognstad et al., 2013).

In hospitalized patients, the poor outcomes achieved by educational interventions can be related to the low interactivity during the education intervention, the lack of knowledge of the clinicians, and the characteristics of the ward included that sometimes make difficult the collection of the data (Franchi et al., 2016).

The implementation of a CDSS in hospitals had a positive impact on 50% of the studies (Urfer et al., 2016; Vanderman et al., 2017). The lack of efficacy of the intervention in the remaining studies can be related to the study design and the fact that the applied criteria are not setting-directed originating a high number of alerts that tend to be ignored by a healthcare professional (McDonald et al., 2019; Winata et al., 2020).

Multifaceted interventions were described as mixed interventions that can reduce the number of PIM (Rahme et al., 2005). In the hospital setting, it was observed that in the two studies that used a combination of educational and medication review strategies, the intervention was well successful (Najjar et al., 2018; Vu and Huong, 2019). In primary care, a combination of educational and medication review strategies results in increased efficacy of the intervention (Clyne et al., 2015; Gibert et al., 2018; Vandenberg et al., 2018).

The results of the included studies suggested that medication review is the most indicated intervention to promote the well-being of the hospitalized patients through the reduction of PIM. The success of medication review strategies at hospital discharge could be related to the fact that the inpatient setting may predispose older adults to new prescriptions and probably unnecessary drugs (Page et al., 2010). Moreover, during the hospitalization physicians tend to resist the change or discontinuation of chronic medication, particularly if the medication is not related to the reason for hospitalization (Page et al., 2010). This high number of prescribed PIM during the hospitalization can be the result of the lack of implemented PIM programs directed to each hospital ward and/or specific condition (Motter et al., 2018).

To improve the well-being of older adults, besides strategies to reduce PIM, strategies to promote appropriate prescription have also been developed. Medication review is a widely used strategy and with better outcomes to reduce potentially inappropriate prescribing (PIP) in hospitalized older adults. However, in a recent review, Dautzenberg et al. (2021) reported that the heterogeneity between studies does not allow reaching significant conclusion. According to dos Santos et al. (2019), the choice of outcome measures, study design, and methodological quality of medication review studies make it difficult to analyze the effectiveness of this strategy. The failure of medication review strategies in primary care can be attributed to the lack of time of physicians to perform the medication review (Plácido et al., 2020); also, as a result of this lack of time, even when the medication review was performed, patients' follow-up did not occur (Campins et al., 2016). On the other hand, educational strategies allow the empowerment of primary care physicians who already had enough handling in managing older adults but not the right confidence and knowledge to manage PIM prescription (Maio et al., 2011). Moreover, educational strategies had more impact on prescribing patterns than presenting a physician only with a decision algorithm (Rahme et al., 2005). A previous work observed similar results regarding the effectiveness of educational strategies to reduce PIP in primary care. According to Kunstler et al. (2019), educational strategies are well successful in changing health professional prescribing behavior.

A recent systematic review focused on non-clinical programs to reduce the inappropriate or unnecessary use of medicines observed that interventions consisting of education messages and recommended behavior alternatives were more likely to be successful in reducing the inappropriate use of medicines or medical procedures (Lin et al., 2020). Educational strategies are essential to improve prescription, as observed by Amorim et al. (2021) since physician-related characteristics can influence the number of PIM prescriptions.

Regarding the multifaceted strategies, the scarcity of studies using this approach did not allow clarifying their benefits in PIM reduction.

In the hospital setting, CDSS interventions significantly reduced the PIM number in older adults. Similar results were found in another systematic review (Dalton et al., 2018). The lack of CDSS in primary care can be related to the outdated user interface model (Price et al., 2017).

Regarding the organizational strategies, the studies achieved a significant impact on pharmacotherapy independently of the setting (Wessell et al., 2008; Stevens et al., 2017).

In 47.8% of the included studies, the intervention was performed by a pharmacist or by a multifaceted team that includes at least one pharmacist. Among these studies, the rate that interventions succeeded well was 72.7%. In the remaining studies, the rate of success observed was 62.5%, suggesting that the inclusion of a pharmacist in the PIM interventions team can be beneficial. Previously it was demonstrated that pharmacists are actively engaged in several care-delivery models such as direct patients care and collaborative team-based care, improving pharmacotherapy and ameliorating the patients-related health outcomes (Lee et al., 2015). It was also reported that pharmacists could play an important role in patients' medication review in practice settings such as community pharmacies long-term care facilities, outpatient clinic home care, and hospitals. Moreover, pharmacists-led deprescribing interventions can reduce the number of unnecessary and potentially harmful medications (Silva et al., 2019; Hernández-Prats et al., 2021).

Although this study was performed with scientific rigors, some limitations are present. The search strategy was limited to the three main health research databases and articles written in English, Portuguese, and Spanish. The included studies were heterogeneous in practice settings, population, size of the samples, and PIM definition that can be variable depending on the screening tool used, which can influence PIM number detected (Thomas and Thomas, 2019; Perpétuo et al., 2021).

Because this review includes studies independently of the quality assessment analysis, an outcomes bias can be aroused. The bias can be attributed to a lack of randomization and blinded interventions and absences/inadequate follow-up period in some studies, compromising a possible scaling up of the interventions.

This study provided valuable data regarding PIM-reduction strategies; however, most of the included studies presented limitations that restrain the extrapolation of the results and a lack of an economic evaluation. Only one study reported that in the intervention group, a significantly lower medication cost was achieved (Frankenthal et al., 2014).

A recent systematic review only found seven articles reporting the economic impact of PIM interventions and suggested that although limited, interventions to optimize medication may outweigh their implementation costs (Laberge et al., 2021).

## Data Availability

The original contributions presented in the study are included in the article/Supplementary Material, further inquiries can be directed to the corresponding author.
