# Supplementary material for: Effectiveness of Interventions to Reduce Potentially Inappropriate Medication in Older Patients: A Systematic Review
Source: Front Pharmacol. 2022 Jan 24;12:777655. doi: 10.3389/fphar.2021.777655 (PMC8819092; doi:10.3389/fphar.2021.777655)
Supplement: Supplementary file 1 [file Table1.DOCX]

Supplementary Material

Supplementary Table S1: PRISMA Checklist

| **Section and Topic** | **Item #** | **Checklist item** | **Location where item is reported** |
| --- | --- | --- | --- |
| **TITLE** | | |  |
| Title | 1 | Identify the report as a systematic review. | 1 |
| **ABSTRACT** | | |  |
| Abstract | 2 | See the PRISMA 2020 for Abstracts checklist. | 1 and 2 |
| **INTRODUCTION** | | |  |
| Rationale | 3 | Describe the rationale for the review in the context of existing knowledge. | 2 |
| Objectives | 4 | Provide an explicit statement of the objective(s) or question(s) the review addresses. | 2 |
| **METHODS** | | |  |
| Eligibility criteria | 5 | Specify the inclusion and exclusion criteria for the review and how studies were grouped for the syntheses. | 3 |
| Information sources | 6 | Specify all databases, registers, websites, organisations, reference lists and other sources searched or consulted to identify studies. Specify the date when each source was last searched or consulted. | 2 and 3 |
| Search strategy | 7 | Present the full search strategies for all databases, registers and websites, including any filters and limits used. | 2 and 3 |
| Selection process | 8 | Specify the methods used to decide whether a study met the inclusion criteria of the review, including how many reviewers screened each record and each report retrieved, whether they worked independently, and if applicable, details of automation tools used in the process. | 3 |
| Data collection process | 9 | Specify the methods used to collect data from reports, including how many reviewers collected data from each report, whether they worked independently, any processes for obtaining or confirming data from study investigators, and if applicable, details of automation tools used in the process. | 3 |
| Data items | 10a | List and define all outcomes for which data were sought. Specify whether all results that were compatible with each outcome domain in each study were sought (e.g. for all measures, time points, analyses), and if not, the methods used to decide which results to collect. | 3 and 4 |
|  | 10b | List and define all other variables for which data were sought (e.g. participant and intervention characteristics, funding sources). Describe any assumptions made about any missing or unclear information. | 3 and 4 |
| Study risk of bias assessment | 11 | Specify the methods used to assess risk of bias in the included studies, including details of the tool(s) used, how many reviewers assessed each study and whether they worked independently, and if applicable, details of automation tools used in the process. | 3 and 4 |
| Effect measures | 12 | Specify for each outcome the effect measure(s) (e.g. risk ratio, mean difference) used in the synthesis or presentation of results. | 4 |
| Synthesis methods | 13a | Describe the processes used to decide which studies were eligible for each synthesis (e.g. tabulating the study intervention characteristics and comparing against the planned groups for each synthesis (item #5)). | 3 |
|  | 13b | Describe any methods required to prepare the data for presentation or synthesis, such as handling of missing summary statistics, or data conversions. | 3 and 4 |
|  | 13c | Describe any methods used to tabulate or visually display results of individual studies and syntheses. | 3 and 4 |
|  | 13d | Describe any methods used to synthesize results and provide a rationale for the choice(s). If meta-analysis was performed, describe the model(s), method(s) to identify the presence and extent of statistical heterogeneity, and software package(s) used. | 3 and 4 |
|  | 13e | Describe any methods used to explore possible causes of heterogeneity among study results (e.g. subgroup analysis, meta-regression). | 4 |
|  | 13f | Describe any sensitivity analyses conducted to assess robustness of the synthesized results. | 3 and 4 |
| Reporting bias assessment | 14 | Describe any methods used to assess risk of bias due to missing results in a synthesis (arising from reporting biases). | 3 |
| Certainty assessment | 15 | Describe any methods used to assess certainty (or confidence) in the body of evidence for an outcome. | - |
| **RESULTS** | | |  |
| Study selection | 16a | Describe the results of the search and selection process, from the number of records identified in the search to the number of studies included in the review, ideally using a flow diagram. | 4 |
|  | 16b | Cite studies that might appear to meet the inclusion criteria, but which were excluded, and explain why they were excluded. | Supplementary Table S1 |
| Study characteristics | 17 | Cite each included study and present its characteristics. | 4, 5 Table 1 and Table 2 |
| Risk of bias in studies | 18 | Present assessments of risk of bias for each included study. | 5 |
| Results of individual studies | 19 | For all outcomes, present, for each study: (a) summary statistics for each group (where appropriate) and (b) an effect estimate and its precision (e.g. confidence/credible interval), ideally using structured tables or plots. | Table 1 and Table 2 |
| Results of syntheses | 20a | For each synthesis, briefly summarise the characteristics and risk of bias among contributing studies. | 5 to 8 |
|  | 20b | Present results of all statistical syntheses conducted. If meta-analysis was done, present for each the summary estimate and its precision (e.g. confidence/credible interval) and measures of statistical heterogeneity. If comparing groups, describe the direction of the effect. | 5 to 8 |
|  | 20c | Present results of all investigations of possible causes of heterogeneity among study results. | - |
|  | 20d | Present results of all sensitivity analyses conducted to assess the robustness of the synthesized results. | 5 |
| Reporting biases | 21 | Present assessments of risk of bias due to missing results (arising from reporting biases) for each synthesis assessed. | - |
| Certainty of evidence | 22 | Present assessments of certainty (or confidence) in the body of evidence for each outcome assessed. | 5 to 8 |
| **DISCUSSION** | | |  |
| Discussion | 23a | Provide a general interpretation of the results in the context of other evidence. | 9 to 11 |
|  | 23b | Discuss any limitations of the evidence included in the review. | 9 to 11 |
|  | 23c | Discuss any limitations of the review processes used. | 11 |
|  | 23d | Discuss implications of the results for practice, policy, and future research. | 11 |
| **OTHER INFORMATION** | | |  |
| Registration and protocol | 24a | Provide registration information for the review, including register name and registration number, or state that the review was not registered. | 2 |
|  | 24b | Indicate where the review protocol can be accessed, or state that a protocol was not prepared. | 2 |
|  | 24c | Describe and explain any amendments to information provided at registration or in the protocol. | - |
| Support | 25 | Describe sources of financial or non-financial support for the review, and the role of the funders or sponsors in the review. | 12 |
| Competing interests | 26 | Declare any competing interests of review authors. | 12 |
| Availability of data, code and other materials | 27 | Report which of the following are publicly available and where they can be found: template data collection forms; data extracted from included studies; data used for all analyses; analytic code; any other materials used in the review. | - |

*From:*  Page MJ, McKenzie JE, Bossuyt PM, Boutron I, Hoffmann TC, Mulrow CD, et al. The PRISMA 2020 statement: an updated guideline for reporting systematic reviews. BMJ 2021;372:n71. doi: 10.1136/bmj.n71

For more information, visit: <http://www.prisma-statement.org/>

**Supplementary Table S2**: Excluded articles with reason (n=~~34~~ 44)

| **No** | **Authors** | **Year** | **Title** | **Reason for exclusion** |
| --- | --- | --- | --- | --- |
| 1 | Balsom et al | 2020 | Impact of a pharmacist‑administered deprescribing intervention on nursing home residents: a randomized controlled trial | Do not address interventions |
| 2 | Chivapricha et al | 2020 | Impact of Geriatric Pharmacy Specialist Interventions to Reduce Potentially Inappropriate Medication Among Hospitalized Elderly Patients at Medical Wards: A Prospective Quasi‑Experimental Study | Do not address older patients (≥65 years old) |
| 3 | Jang et al | 2020 | Impact of a nationwide prospective drug utilization review program to improve prescribing safety of potentially inappropriate medications in older adults: An interrupted time series with segmented regression analysis | Addresses a pre-selected and/or a limited number of medicines |
| 4 | Gedde et al | 2020 | Less Is More: The Impact of Deprescribing Psychotropic Drugs on Behavioral and Psychological Symptoms and Daily Functioning in Nursing Home Patients. Results From the Cluster-Randomized Controlled COSMOS Trial | Did not report PIM specific outcomes |
| 5 | Houlind et al | 2020 | A Collaborative Medication Review Including Deprescribing for Older Patients in an Emergency Department: A Longitudinal Feasibility Study | Did not report PIM specific outcomes |
| 6 | Leguelinel-Blache et al | 2020 | Impact of pharmacist-led multidisciplinary medication review on the safety and medication cost of the elderly people living in a nursing home: a before-after study | Did not report PIM specific outcomes |
| 7 | Schapira et al | 2020 | A multifactorial intervention to lower potentially inappropriate medication use in older adults in Argentina | Addresses a pre-selected and/or a limited number of medicines |
| 8 | Rieckert et al | 2020 | Use of an electronic decision support tool to reduce polypharmacy in elderly people with chronic diseases: cluster randomised controlled trial | Did not report PIM specific outcomes |
| 9 | Alosaimy et al | 2019 | Effect of a Pharmacist‑Driven Medication Management Intervention Among Older Adults in an Inpatient Setting | Do not address interventions |
| 10 | Cossette et al | 2019 | A pharmacist-physician intervention model using a computerized alert system to reduce high-risk medication use in primary care | Addresses a pre-selected and/or a limited number of medicines |
| 11 | Parker et al | 2019 | Effectiveness of using STOPP/START criteria to identify potentially inappropriate medication in people aged ≥65 years with chronic kidney disease: a randomized clinical trial | Addresses PIM in patient-specific diseases |
| 12 | Prasert et al | 2019 | Effect of a computerized decision support system on potentially inappropriate medication prescriptions for elderly patients in Thailand | Do not address older patients (≥65 years old) |
| 13 | Santolaya-Perrín et al | 2019 | The efficacy of a medication review programme conducted in an emergency department | Did not report PIM specific outcomes |
| 14 | Sanz-Tamargo et al | 2019 | Adaptation of a deprescription intervention to the medication management of older people living in long-term care facilities | Did not report PIM specific outcomes |
| 15 | Blanc et al | 2018 | Prevention of potentially inappropriate medication in internal medicine patients: A prospective study using the electronic application PIM-Check | Did not report PIM specific outcomes |
| 16 | Cool et al | 2018 | Reducing potentially inappropriate drug prescribing in nursing home residents: effectiveness of a geriatric intervention | Do not address older patients (≥65 years old) |
| 17 | Martin et al | 2018 | Effect of a Pharmacist-Led Educational Intervention on Inappropriate Medication Prescriptions in Older Adults The D-PRESCRIBE Randomized Clinical Trial | Addresses a pre-selected and/or a limited number of medicines |
| 18 | Whitman et al | 2018 | Pharmacist-led medication assessment and deprescribing intervention for older adults with cancer and polypharmacy: a pilot study | Addresses PIM in patient-specific diseases |
| 19 | Cossette et al | 2017 | Reduction in targeted potentially inappropriate medication use in elderly inpatients: a pragmatic randomized controlled trial | Addresses a pre-selected and/or a limited number of medicines |
| 20 | Fried et al | 2017 | Effect of the Tool to Reduce Inappropriate Medications on Medication Communication and Deprescribing | Did not report PIM specific outcomes |
| 21 | Lagrange et al | 2017 | A context-aware decision-support system in clinical pharmacy: Drug monitoring in the elderly | Did not report PIM specific outcomes |
| 22 | Lenander et al | 2017 | Effects of an intervention (SÄKLÄK) on prescription of potentially inappropriate medication in elderly patients | Addresses a pre-selected and/or a limited number of medicines |
| 23 | Cossette et al | 2016 | Knowledge Translation Strategy to Reduce the Use of Potentially Inappropriate Medications in Hospitalized Elderly Adults | Addresses a pre-selected and/or a limited number of medicines |
| 24 | Deliens et al | 2016 | Drugs prescribed for patients hospitalized in a geriatric oncology unit: Potentially inappropriate medications and impact of a clinical pharmacist | Addresses PIM in patient-specific diseases |
| 25 | Kimura et al | 2016 | Potentially inappropriate medications in elderly Japanese patients: effects of pharmacists’ assessment and intervention based on Screening Tool of Older Persons’ Potentially Inappropriate Prescriptions criteria ver.2 | Do not address interventions |
| 26 | Mudge et al | 2016 | Effects of a pilot multidisciplinary clinic for frequent attending elderly patients on deprescribing | Do not address older patients (≥65 years old) |
| 27 | Arvisais et al | 2015 | A Pharmacist–Physician Intervention Model Using a Computerized Alert System to Reduce High-Risk Medication Use in Elderly Inpatients | Addresses a pre-selected and/or a limited number of medicines |
| 28 | Alassaad et al | 2014 | The effects of pharmacist intervention on emergency department visits in patients 80 years and older: subgroup analyses by number of prescribed drugs and appropriate prescribing | Did not report PIM specific outcomes |
| 29 | Olson et al | 2014 | Optimization of Decision Support Tool using Medication Regimens to Assess Rehospitalization Risks | Did not report PIM specific outcomes |
| 30 | O’Sullivan et al | 2014 | The Impact of a Structured Pharmacist Intervention on the Appropriateness of Prescribing in Older Hospitalized Patients | Did not report PIM specific outcomes |
| 31 | Peterson et al | 2014 | Electronic Surveillance and Pharmacist Intervention for Vulnerable Older Inpatients on High-Risk Medication Regimens | Addresses a pre-selected and/or a limited number of medicines |
| 32 | Milos et al | 2013 | Improving the Quality of Pharmacotherapy in Elderly Primary Care Patients Through Medication Reviews: A Randomised Controlled Study | Addresses a pre-selected and/or a limited number of medicines |
| 33 | Roughead et al | 2013 | Bridging evidence-practice gaps: improving use of medicines in elderly Australian veterans | Did not report PIM specific outcomes |
| 34 | Lang et al | 2012 | Interdisciplinary Geriatric and Psychiatric Care Reduces Potentially Inappropriate Prescribing in the Hospital: Interventional Study in 150 Acutely Ill Elderly Patients with Mental and Somatic Comorbid Conditions | Addresses PIM in patient-specific diseases |
| 35 | Thorpe et al | 2012 | The Impact of Family Caregivers on Potentially Inappropriate Medication Use in Noninstitutionalized Older Adults With Dementia | Do not address interventions |
| 36 | Beer et al | 2011 | A pilot randomized controlled trial of deprescribing | Do not address interventions |
| 37 | Lapane et al | 2011 | Evaluation of the Fleetwood Model of Long-Term Care Pharmacy | Addresses a pre-selected and/or a limited number of medicines |
| 38 | Mattison et al | 2010 | Preventing Potentially Inappropriate Medication Use in Hospitalized Older Patients With a Computerized Provider Order Entry Warning System | Addresses a pre-selected and/or a limited number of medicines |
| 39 | Stuijt et al | 2008 | Appropriateness of Prescribing among Elderly Patients in a Dutch Residential Home | Did not report PIM specific outcomes |
| 40 | Raebel et al | 2007 | Randomized Trial to Improve Prescribing Safety in Ambulatory Elderly Patients | Addresses a pre-selected and/or a limited number of medicines |
| 41 | Smith et al | 2006 | The Impact of Prescribing Safety Alerts for Elderly Persons in an Electronic Medical Record | Did not report PIM specific outcomes |
| 42 | Briesacher et al | 2005 | Evaluation of Nationally Mandated Drug Use Reviews to Improve Patient Safety in Nursing Homes: A Natural Experiment | Do not address older patients (≥65 years old) |
| 43 | Lane et al | 2004 | Potentially Inappropriate Prescribing in Ontario Community-Dwelling Older Adults and Nursing Home Residents | Addresses a pre-selected and/or a limited number of medicines |
| 44 | Harlacher et al | 2000 | Geriatric assessment in the elderly cancer patient | Do not address interventions |
